# Deep learning-based histopathological segmentation for whole slide images of colorectal cancer in a compressed domain

**DOI:** 10.1038/s41598-021-01905-z

**Published:** 2021-11-18

**Authors:** Hyeongsub Kim, Hongjoon Yoon, Nishant Thakur, Gyoyeon Hwang, Eun Jung Lee, Chulhong Kim, Yosep Chong

**Affiliations:** 1 Departments of Electrical Engineering, Creative IT Engineering, Mechanical Engineering, School of Interdisciplinary Bioscience and Bioengineering, Medical Device Innovation Center, and Graduate School of Artificial Intelligence, Pohang University of Science and Technology (POSTECH), Pohang, 37674 South Korea; 2 Deepnoid Inc., Seoul, 08376 South Korea; 3 Department of Hospital Pathology, The Catholic University of Korea, College of Medicine, Uijeongbu St. Mary’s Hospital, Seoul, South Korea; 4 Department of Hospital Pathology, The Catholic University of Korea, College of Medicine, Yeouido St. Mary’s Hospital, Seoul, South Korea; 5 Department of Pathology, Shinwon Medical Foundation, Gwangmyeong-si, Gyeonggi-do, South Korea

**Keywords:** Computer science, Image processing, Machine learning

## Abstract

Automatic pattern recognition using deep learning techniques has become increasingly important. Unfortunately, due to limited system memory, general preprocessing methods for high-resolution images in the spatial domain can lose important data information such as high-frequency information and the region of interest. To overcome these limitations, we propose an image segmentation approach in the compressed domain based on principal component analysis (PCA) and discrete wavelet transform (DWT). After inference for each tile using neural networks, a whole prediction image was reconstructed by wavelet weighted ensemble (WWE) based on inverse discrete wavelet transform (IDWT). The training and validation were performed using 351 colorectal biopsy specimens, which were pathologically confirmed by two pathologists. For 39 test datasets, the average Dice score, the pixel accuracy, and the Jaccard score were 0.804 ± 0.125, 0.957 ± 0.025, and 0.690 ± 0.174, respectively. We can train the networks for the high-resolution image with the large region of interest compared to the result in the low-resolution and the small region of interest in the spatial domain. The average Dice score, pixel accuracy, and Jaccard score are significantly increased by 2.7%, 0.9%, and 2.7%, respectively. We believe that our approach has great potential for accurate diagnosis.

## Introduction

The large number of inspections for pathologists is exposed to the risk of misdiagnosis. This leads to a rapid increase in medical expenses, an increase in the false diagnosis rate, a decrease in medical productivity, and the risk of a cancer diagnosis. Automatic analyses of pathological images can mitigate human effort, save time, and provide a confident foundation for surgery and treatment. Convolutional neural networks (CNNs) are especially popular for the automatic diagnosis of many diseases in pathology^1–19^. However, despite the continued increase in the speed and memory capacity of central processing units (CPUs) and graphical processing units (GPUs), technological advances in pathological image analysis are still hampered by large image sizes^20^.

For high-resolution and large-scale images, a general preprocessing method to relieve memory limitation can induce important information loss. Several methods have been explored to reduce image sizes, such as decimation, cropping, and compression^21–23^. Decimation is the major process for down sampling large images, and it can also reduce noise power and improve signal-to-noise ratios (SNRs), thanks to an anti-aliasing filter. However, decimation can cause a loss of high-frequency information, resulting in low resolution due to the reduced signal bandwidth^24,25^. As another widely used method, cropping extracts the wanted areas from whole slide images (WSIs) into tiles. Although no information is missed with respect to a single tile, the spatial relationships between tiles may be lose, which is critical because object judgments depend on the relative size and color of each cell in the pathological image.

Compression is widely used both to minimize the size of an image file without degradation in the image quality and to reduce irrelevance and redundancy of data in the image. Thus, compression is mostly preferred to process large-scale images. For example, detecting ships in satellite images is difficult due to their high resolution and correspondingly large data volume. A compression technique called discrete wavelet transform (DWT) resolves the difficulty in high-resolution ship detection and performs better than conventional computer vision algorithms^26^. In addition, DWT is also useful for texture classification, because its finite duration provides both the frequency and spatial locality. In pathology, DWT analysis has been applied to classify tumors by using texture analysis^27^.

In this work, we propose a pathological image segmentation method in the compressed domain. To compress large pathological images, we utilized not only DWT but also principal component analysis (PCA) according to hematoxylin and eosin (H&E) staining characteristics to reduce 3-channel RGB data to one channel^28^. We tested this inference method in the compressed domain on colorectal cancer pathologic images from the Catholic University of Korea Yeouido St. Mary’s Hospital.

Our results imply that the method using the compressed domain is more useful for pathologic segmentation than the method using the spatial domain, for three reasons: (1) The average Dice score, pixel accuracy, and Jaccard score are significantly improved, by 2.7%, 0.9%, and 2.7%, respectively. (2) Using DWT, neural networks can be trained not only by spatial information but also by texture information. (3) The performance can be more robust because of the large ROI in training after compression; the size of the input image is reduced by 8%. This new segmentation technique in the compressed domain can be potentially useful in applications where large-scale data and texture information are important, such as remote sensing^29^ and microscopy^30–32^.

## Results

### Data distribution

We used 390 WSIs of colorectal biopsy specimens. The average size of WSIs was 43,443 by 28,645 pixels. We split the dataset into two groups: 351 train and validation data, and 39 test data (Supplementary Table 1). We used this dataset to implement a pipeline to achieve binary segmentation of normal and abnormal areas in colorectal cancer (CRC) tissue images.

### Overall result according to each method

Table 1 compares the average the Dice score (Dice), pixel accuracy (Acc), and Jaccard score (Jac) according to each method. As it shows, for the model using information loss data (Small ROI, low resolution), the average Dice, Acc, and Jac results decreased by 1.1% , 0.2%, and 1.2% for the small ROI data (Tile size: 256 by 256, 20× magnification) and 4%, 1%, and 4.4% for the low resolution data (Tile size: 512 by 512, 10× magnification) respectively, compared to those of the model using standard data (Tile size: 512 by 512, 20× magnification). For the model using compressed data, the average Dice, Acc, and Jac results for the LL sub-band increased by 4%, 0.6%, and 4.3%, respectively, compared to those of the model using low resolution data in spatial domain whose magnification equal to LL sub-band’s magnification. The reason why LL's results improve is the impact of PCA. Channels are reduced and background is removed, reducing input complexity and improving performance. However, the average Dice and Acc results of the LH (0.1% for Dice, -0.7% for Acc, and − 0.5% for Jac), HL (− 1.8% for Dice, − 0.3% for Acc, and − 3.6% for Jac), and HH (− 1.3% for Dice, − 0.8% for Acc, and − 2.5% for Jac) sub-bands carrying high-frequency information decreased compared to those before compression. For the result of ensemble method, the average Dice, Acc, and Jac results for wavelet weighted ensemble (WWE) result for wavelet sub-band after principal component analysis (PCA) increased by 2.7%, 0.9%, and 2.7%, respectively, compared to those of the model using standard data in spatial domain. It is best performance among our ensemble method.

**Table 1:** Average Dice, Acc, and Jac values for the result of standard input in spatial domain (Tile size: 512 by 512, ×20 magnification, standard), the result of small ROI input in spatial domain (Tile size: 256 by 256, ×20 magnification, Small ROI), the result of low resolution input in spatial domain (Tile size: 512 by 512, ×10 magnification, low resolution), the result of weighted average ensemble (WAE) result for wavelet sub-band after grayscale conversion (GRAY-DWT (WAE)), the result of wavelet weighted ensemble (WWE) result for wavelet sub-band after grayscale conversion (GRAY-DWT (WWE)), the result of weighted average ensemble (WAE) result for wavelet sub-band after principle component analysis (PCA) (PCA-DWT (WAE)), and the result of wavelet weighted ensemble (WWE) result for wavelet sub-band after principal component analysis (PCA) (PCA-DWT (WWE)).

Method		Dice	Acc	Jac
Spatial domain	Standard	0.777 ± 0.133	0.948 ± 0.030	0.663 ± 0.186
	Small ROI	0.766 ± 0.146	0.946 ± 0.032	0.651 ± 0.199
	Low resolution	0.737 ± 0.164	0.938 ± 0.019	0.619 ± 0.197
Compressed domain	LL sub-band	0.777 ± 0.157	0.944 ± 0.037	0.662 ± 0.206
	LH sub-band	0.738 ± 0.172	0.931 ± 0.040	0.614 ± 0.218
	HL sub-band	0.719 ± 0.153	0.935 ± 0.030	0.583 ± 0.184
	HH sub-band	0.724 ± 0.167	0.929 ± 0.038	0.594 ± 0.206
Ensemble	GRAY-DWT (WAE)	0.782 ± 0.155	0.944 ± 0.042	0.668 ± 0.207
	GRAY-DWT (WWE)	0.790 ± 0.131	0.949 ± 0.027	0.671 ± 0.177
	PCA-DWT (WAE)	0.785 ± 0.159	0.947 ± 0.040	0.674 ± 0.208
	PCA-DWT (WWE)	0.804 ± 0.125	0.957 ± 0.025	0.690 ± 0.174

### The trend for dice according to each class

**Figure 1:**
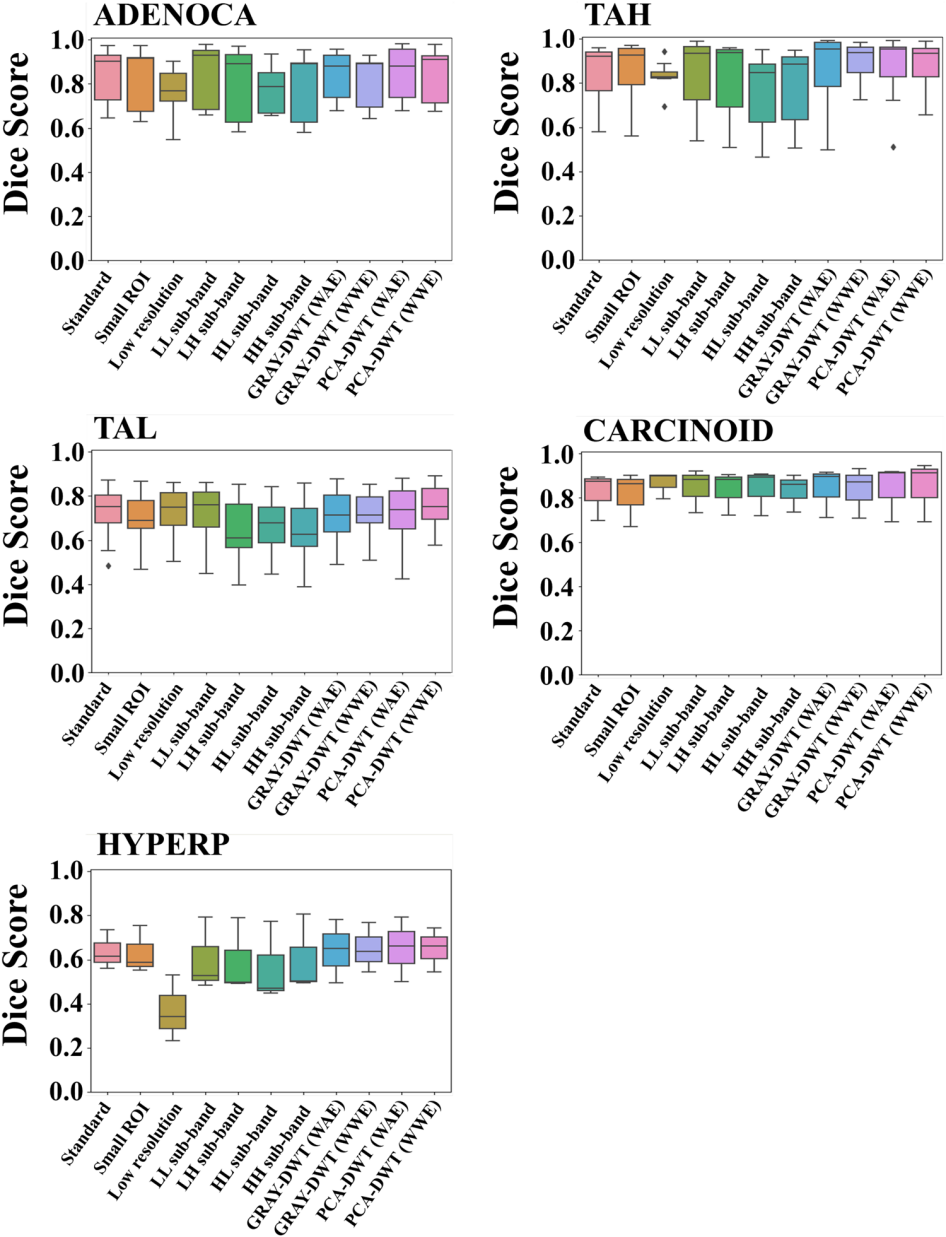
Comparison of Dice for each class between the result of standard input in spatial domain (Tile size: 512 by 512, ×20 magnification, standard), the result of small ROI input in spatial domain (Tile size: 256 by 256, ×20 magnification, Small ROI), the result of low resolution input in spatial domain (Tile size: 512 by 512, ×10 magnification, low resolution), the result of weighted average ensemble (WAE) result for wavelet sub-band after grayscale conversion (GRAY-DWT (WAE)), the result of wavelet weighted ensemble (WWE) result for wavelet sub-band after grayscale conversion (GRAY-DWT (WWE)), the result of weighted average ensemble (WAE) result for wavelet sub-band after principle component analysis (PCA) (PCA-DWT (WAE)), and the result of wavelet weighted ensemble (WWE) result for wavelet sub-band after principal component analysis (PCA) (PCA-DWT (WWE)).

Figure 1 shows distribution Dice according to all classes. In the case of all tumor classes, the average results for the LL sub-band are relatively high. Further, the average results of the LH, HL, HH sub-bands carrying high-frequency components are relatively high in ADENOCA, TAH, CARCINOID, and HYPERP (Fig. 1). ADENOCA (malignant tumors occurring in the mucosa), TAH (relatively high advanced), CARCINOID (malignant tumors but occurring in the submucosa), and HYPERP (benign tumors) (Fig. 1), which are relatively easy to detect due to advanced disease progression and consequent pathological modifications. However, the results of the LH, HL, HH sub-bands are less predictive for TAL (Fig. 1). TAL (relatively less advanced) are difficult to accurately predict with only high-frequency components. Based on these results, we propose an ensemble method that can improve the results using both low-frequency and high-frequency information. Compared to the no compression results, ADENOCA, TAH, CARCINOID, and HYPERP show good performance after WAE because the Dice in the high-frequency sub-bands such as LH, HL, and HH sub-band are higher than these of the no compression case (ADENOCA: + 3.3% for Dice at PCA-DWT(WAE); TAH: + 1.9% for Dice at PCA-DWT(WAE); CARCINOID: + 1.8% for Dice at PCA-DWT(WAE); HYPERP: + 1.4% for Dice at PCA-DWT(WAE)). However, in TAL, which show low performance in the high-frequency sub-bands, the Dice after PCA-DWT(WAE) are lower than those of no compression (TAL: − 1.5% for Dice at PCA-DWT(WAE)). On the other hand, after PCA-DWT(WWE), the average Dice increase by about 2.7%, respectively, compared to LL. For each class, the results of ADENOCA (− 1.0% for Dice), TAH (+ 1.3% for Dice), TAL (+ 4.3% for Dice), CARCINOID (+ 0.9% for Dice), and HYPER (− 0.1% for Dice) gradually increase.

### Change in dice in all classes according to low- (${{W}}_{1}$) and high-frequency weight (${{W}}_{2}$, ${{W}}_{3}$, and ${{W}}_{4}$)

**Figure 2:**
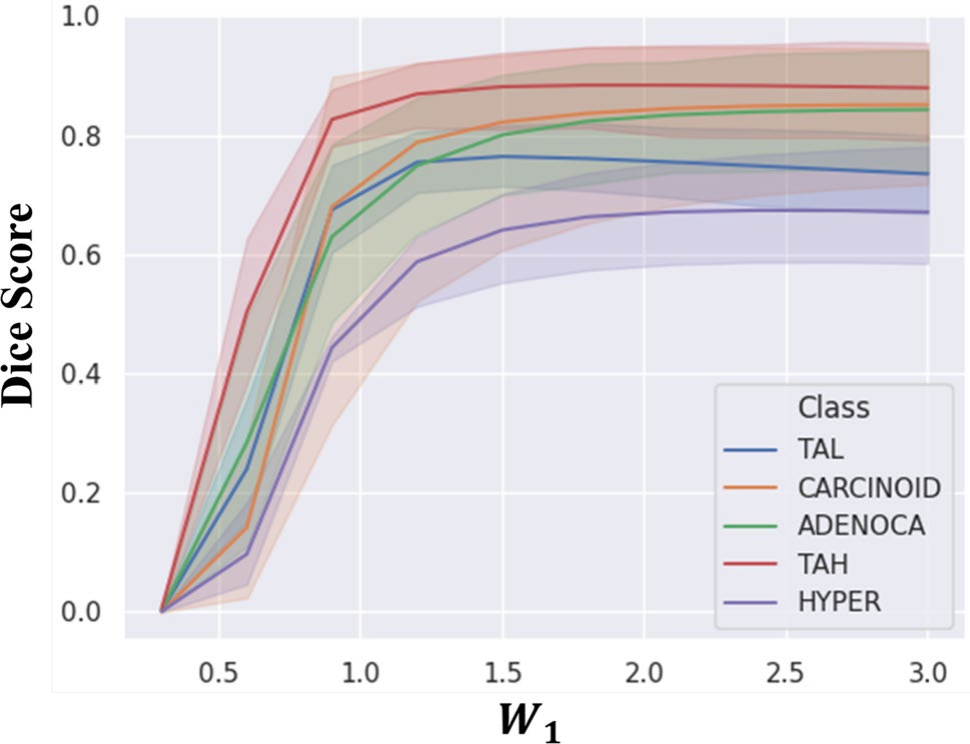
Change in Dice in all tumor classes according to the low-frequency weight (${W}_{1}$) in the WWE.

We checked change of Dice score in all classes according to low-frequency weight (${W}_{1}$) and high-frequency weight (${W}_{2}$, ${W}_{3}$, and ${W}_{4}$) to optimize each weight by conducting the empirical test. The best weights in the WWE are determined by the average Dice scores, as shown in Supplementary Table 2. Figure 2 describes the change in Dice score with respect to various low-frequency weights (${W}_{1}$) in all tumor classes (ADENOCA, TAH, TAL, CARCINOID, and HYPERP). From 0.3 to 0.9, the Dice scores of all the classes increase relatively steeply. Particularly, the increasing rates in the Dice scores of HYPERP and ADENOCA are relatively high. Beyond the ${W}_{1}$ value of 1.5, the Dice scores start being saturated in all classes. Further, we changed the values of the high-frequency weights (${W}_{2}$, ${W}_{3}$, and ${W}_{4}$), but the changes in Dice scores are negligible as shown in Supplementary Fig. 1.

### Comparison of the heat map and line profiles between annotation, the result in the spatial domain and compressed domain

**Figure 3:**
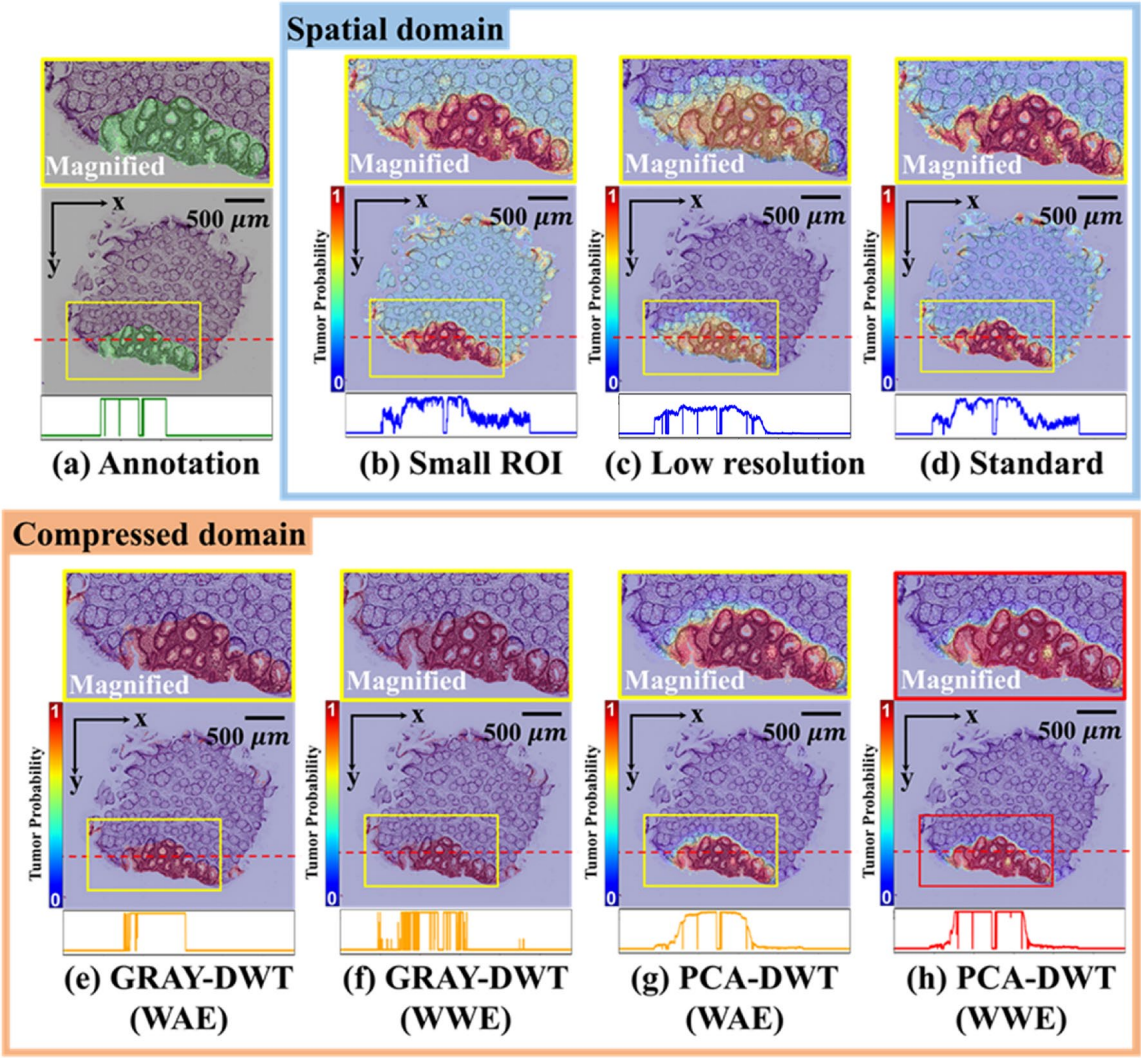
(**a**) Annotation: Annotated image, corresponding magnified image (above), and Dashed red line profiles (below). (**b**) Small ROI: The result of small ROI input in spatial domain (Tile size: 256 by 256, ×20 magnification). (**c**) Low resolution: The result of low-resolution input in spatial domain (Tile size: 512 by 512, ×10 magnification). (**d**) Standard: The result of standard input in spatial domain (Tile size: 512 by 512, ×20 magnification). (**e**) GRAY-DWT (WAE): The result of weighted average ensemble (WAE) result for wavelet sub-band after grayscale conversion. (**f**) GRAY-DWT (WWE): The result of wavelet weighted ensemble (WWE) result for wavelet sub-band after grayscale conversion. (**g**) PCA-DWT (WAE): The result of weighted average ensemble (WAE) result for wavelet sub-band after principle component analysis (PCA). (**f**) PCA-DWT (WWE): The result of wavelet weighted ensemble (WWE) result for wavelet sub-band after principal component analysis (PCA).

Using a heat map and line profiles for tumor probability, we compared the segmentation prediction for annotation, the result in spatial domain, and the result in compressed domain (Fig. 3a–h). The color bar indicates the tumor probability for each pixel. The heat map is overlaid on the original histology image, and a magnified image of the area in the colored border is located on top of the main image. The line profiles of the tumor probability cut along the red dotted dashed lines are located below the main image. Figure 3a is the ground truth, annotated by a pathologist. The pixel value in the annotation is 1, and the value in the other regions is 0. Figure 3b shows the segmentation result of the model using small ROI input data in spatial domain (Tile size: 256 by 256, 20× magnification). There is a slight loss of spatial information after small size tile extraction for efficient training, but the magnification is the same as for the standard methods. Figure 3c shows the segmentation result of the model using low resolution input data in spatial domain (Tile size: 512 by 512, 10× magnification). There is a slight loss of high-frequency information after decimation for efficient training, but the ROI used in single training is the same as for the other methods. Figure 3d shows the segmentation result of the model using standard input data in spatial domain (Tile size: 512 by 512, 20× magnification). Figure 3e shows the segmentation result of weighted average ensemble (WAE) result for wavelet sub-band after grayscale conversion (initial tile size: 1024 by 1024, 20× magnification). Figure 3f shows the segmentation result of wavelet weighted ensemble (WWE) result for wavelet sub-band after grayscale conversion (initial tile size: 1024 by 1024, 20× magnification). Figure 3g shows the segmentation result of weighted average ensemble (WAE) result for wavelet sub-band after PCA (initial tile size: 1024 by 1024, 20× magnification). Figure 3h shows the segmentation result of wavelet weighted ensemble (WWE) result for wavelet sub-band after PCA (initial tile size: 1024 by 1024, 20× magnification). The magnified image in Fig. 3b–g. predicts a broader region than in the annotation, and the tumor probability in each pixel is relatively low. The segmentation result for PCA-DWT(WAE), shown in Fig. 3h, clearly is qualitatively better than that in spatial domain. The final segmentation result with WWE has accurate edges as well as a high probability in each pixel, compared to the other methods. The tumor probability line profile processed with PCA-DWT (WWE) is most similar to the original annotation profile, proving the accuracy of our method.

### Average dice for each method according to the threshold

**Figure 4:**
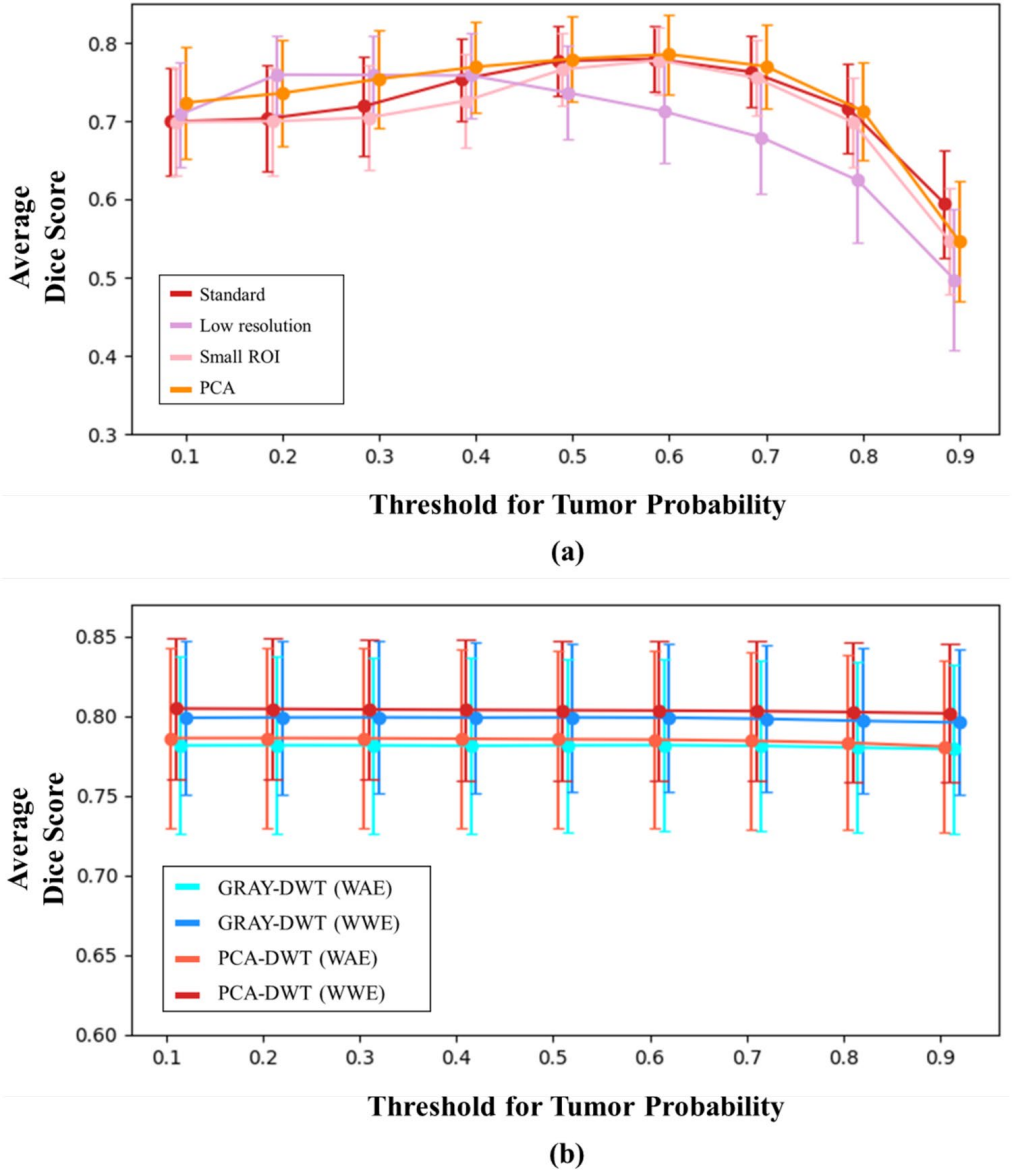
Comparative Dice of wavelet weighted ensemble (WWE) for wavelet sub-bands, weighted average ensemble (WAE) for wavelet sub-bands, and the result in spatial domain according to the threshold for tumor probability (95% confidence interval).

The Dice scores for the result in spatial domain are compared across a range of threshold tumor probability values (Fig. 4a), and WWE for the wavelet sub-bands after PCA or grayscale conversion, WAE for the wavelet sub-bands after PCA or grayscale conversion (Fig. 4b). Between threshold values of 0.1 and 0.6, the Dice score of the result in spatial domain is relatively stable. However, beyond a threshold of 0.7, the Dice score for this method drops sharply, compared to those of the other methods. WAE and WWE continue perform robustly for all thresholds, and the Dice score of WWE is consistently higher than that of WAE, thanks to the high-frequency information.

### Final prediction result of five different tumor classes using PCA-DWT (WWE)

**Figure 5:**
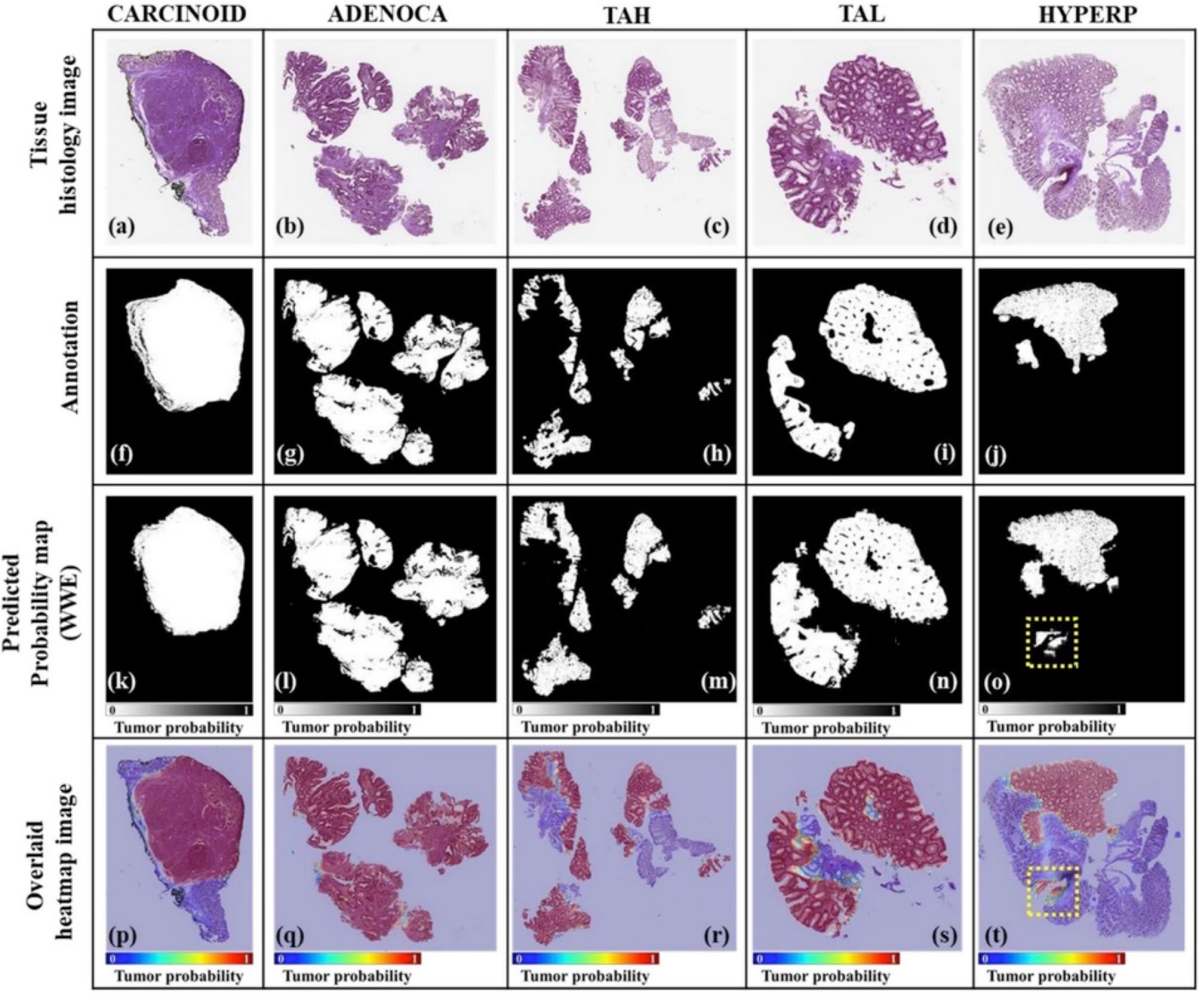
Prediction results of the five different tumor classes. (**a**)–(**e**) are tissue histology images. (**f**)–(**j**) are annotation by a pathologist (i.e., ground truth). (**k**)–(**o**) are predicted probability map (WWE). (**p**)–(**t**) are overlaid tissue histology image and prediction heatmap. Yellow dotted boxes in (**o**) and (**t**) show misprediction due to dead nuclei.

Finally, we compared our PCA-DWT (WWE) predicted image with the image annotated by a pathologist. Figure 5a–e shows tissue histology images from five different tumor categories. The pathologist’s annotations are shown in Fig. 5f–j. The corresponding predicted probability map using PCA-DWT (WWE) are shown in Fig. 5k–o and final overlaid tissue images are shown in Fig. 5p–t. The proposed PCA-DWT (WWE) method generally segmented an afflicted area that corresponded well to the ground truth images. The average Dice, Acc, and Jac of the PCA-DWT (WWE) are 0.802 ± 0.125, 0.957 ± 0.025, and 0.690 ± 0.174 respectively. The best Dice (0.867 ± 0.144) is achieved in TAH, where the high-frequency information is important. On the other hand, the worst Dice (0.652 ± 0.119) is in HYPERP, where the low-frequency information is important. As shown in the yellow dotted boxes in the case of HYPERP (Fig. 5o,t), we often observed that the normal region where dead nuclei are gathered is abnormally predicted. Possibly, these abnormal predictions are caused by artifacts, such as tissue folds, ink, dust, and air bubbles, and further artifact removal may be required. Despite these abnormalities, the overall prediction of colorectal cancer using PCA-DWT(WWE) was not biased to any one class: it performed well for all.

## Discussion

The goal of this study is to increase diagnostic accuracy (e.g., Dice, Acc, and Jac) by using a compressed domain to reduce high-frequency information loss. The compressed domain approach was employed in previous studies^26,33,34^ showing good performance in pathology classification not segmentation because there was no appropriate ensemble method for results for each sub-band (e.g., LL, LH, HL, HH sub-bands results)^27,35–38^. In this paper, we proposed the PCA-DWT(WWE) method, which learns each low-frequency component and high-frequency component in the compression domain and then combines them. With the NVIDIA TITAN X 12 Gb GPU used in this experiment, the U**-**net++ model can be trained on a maximum tile size of 512 by 512 at once. Therefore, in order to learn our experimental ROI size of $6.25 \times {10}^{-2} \; {\upmu {\text{m}} }^{2}$ without compression, the resolution of the standard image (20× magnification) would have to be lowered (10× magnification) (Table 2). In this process, the loss of high-frequency components cannot be avoided. On the other hand, our proposed method can handle a tile size of 1024 by 1024 before compression The main reason why WWE is better than WAE could be WWE gives weights in units of pixels based on wavelet transform, a compression method, according to the characteristics of input data while WAE gives weight in units of images. Thus, it is not necessary to lower the resolution to learn the same ROI size, and learning is possible with 20× magnification. In addition, compared to the result in spatial domain, our proposed method can learn a tile that is four times larger than the limit of the hardware. However, our method requires four times more the number of GPUs (Table 2) at the same time. From the perspective of time resources, in the case of a general CNN based on 2D convolution, the amount of computation increases exponentially as the input size increases. Therefore, it is faster to learn by separating one image into four images than to learn an image that is 4 times larger at a single time. This case is similar to the principle of the Cooley–Tukey FFT algorithm^39,40^, and we believe that subsequent studies will also meaningfully to reduce time consuming.

**Table 2:** The conditions of input image such as magnification, initial tile size, ROI size, and the number of GPUs.

	Spatial domain			Compressed domain
	Low resolution	Standard	Small ROI	
Magnification	** ×10**	×20	×20	×20
Initial tile size	512 by 512	512 by 512	**256 by 256**	1024 by 1024
ROI size	6.25 × 10^–2^ µm^2^	1.56 × 10^–2^ µm^2^	**3.91 × 10**^**–3**^** µm**^**2**^	6.25 × 10^–2^ µm^2^
Number of GPUs	1	1	1	**4**

Drawbacks in each domain are in bold.

Significance values are in bold.

We have conducted a study to prevent the loss of high-frequency information that occurs in the process of having to resize the image due to the limitation of the hardware and to increase the accuracy of the final result by using protected high-frequency information. Using a wavelet-weighted ensemble method, we found that accuracy was improved over that of images in spatial domain. The overall accuracy was determined by the low-frequency component, and the high-frequency component affected the margin. The disadvantage is that it requires a relatively large amount of GPU resources. However, we expect to reduce time-consuming compared to the result in spatial domain when the same as the initial tile size. As for the possible shortcomings of the proposed work, the weights for each frequency should be changed from experimental parameter to trainable parameter. Furthermore, it is difficult to implement explainable AI because our approaches are based on the pre-processing and modified ensemble. To the best of our knowledge, this is the first study to do WWE in the compressed domain. We applied this processing method to colorectal cancer pathology images, and we believe that it can also be applied in general pathology images and show a similar increase in accuracy. Our proposed wavelet-weighted ensemble method can also be applied in other fields that process large-scale images (e.g., astronomy and satellite imagery) and that is important to margin (e.g., radiation therapy).

## Methods

### Data preparation

This study was reviewed and approved by the Institutional Review Board of the Catholic University of Korea College of Medicine (SC18RNSI0116). All experiments were conducted in accordance with relevant guidelines/regulations in the Catholic University of Korea College of Medicine. Informed consent prior to the surgical procedures, all patients had given their informed consent to use tissue samples and pathological diagnostic reports for research purposes. We used a dataset using H&E stained-WSIs of colorectal biopsy specimens at the Yeouido St. Mary’s Hospital.

First, the reason why colorectal cancer (CRC) was chosen is, it is second leading cause of mortality throughout the globe^41,42^. Due to the rapid adaptation of urban lifestyle, it is expected to increase the CRC cases in Asian countries^43^. Early diagnosis is a critical step to minimize the CRC causing death and colonoscopy is one of the powerful screening methods^44^. Moreover, according to Korean health policy, it is recommended that every citizen should undergo a colonoscopy and that leads to higher number of colonoscopy cases. In our hospital, we had higher sample availability of CRC as compared to other cancer.

Another reason is that the histological staining and pathological examination are more time-consuming and labor-intensive work^45^. The pathological diagnosis of CRC samples can be easily influenced by independent pathologists’, their knowledge and experience. It may cause inter-observer and intra-observer variations among pathologists^45^. Currently, there are two types of pathological diagnosis of CRC such as Vienna classification (followed by Western countries) and Japanese classification (followed by Eastern countries)^46,47^. Hence, there is a high urge for a standardized system that can mitigate the confusion among specialists.

The WSIs were 20× magnified images taken using a digital whole-slide camera (Aperio AT2, Leica biosystems, USA). The Whole slide images (WSIs) were manually annotated by the three trained pathologists supervised by the expert and performed routine histopathological examination by drawing the region of interest in the slides that corresponded to one of the five labels: adenocarcinoma (ADENOCA), high-grade adenoma with dysplasia (TAH), and low-grade adenoma with dysplasia (TAL), carcinoid (CARCINOID), and hyperplastic polyp (HYPERP). The average annotation time per WSI took 5–10 min. Next, annotations carried out by the trained pathologists were reviewed by the three senior pathologists and if necessary then modified and verified with the final checking verification by the one senior professors. Cases that had discrepancies in the annotation labels resolved the issue through further discussions. The images were excluded, when it was not possible to reach a consensus on a lesion type for an image. Most of the WSI contained multiple annotation labels. Therefore, a single WSI label of major diagnosis was assigned to a given WSI.

### Compressed image analysis

**Figure 6:**
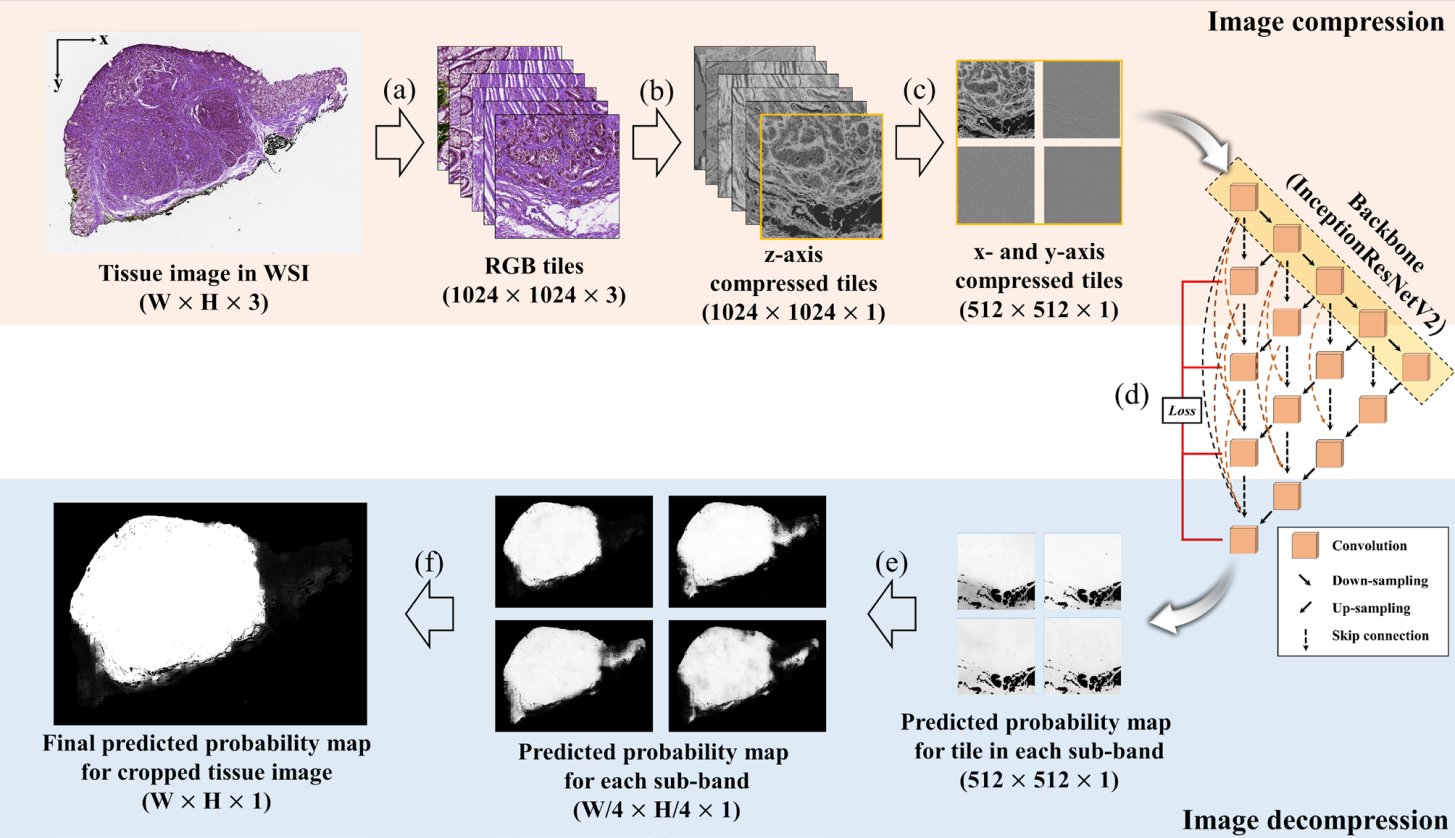
Overall flow chart of the proposed method. (**a**) Tile extraction based on a sliding window. (**b**) Image depth compression. (**c**) Forward transform to the compressed domain. (**d**) Training and prediction using convolutional neural networks. (**e**) Prediction from one tile to the whole image. (**f**) Wavelet-weighted ensemble (WWE).

In this study, we applied a compressed domain based on the wavelet transform used in JPEG2000 for the segmentation of pathologic images. The pipeline is as follows: tile extraction, z-axis compression, training and prediction in the compressed-domain using CNNs, prediction from one tile to the whole image, and wavelet-weighted ensemble (WWE) (Fig. 6). Each process is detailed in the following subsections.

### Tile extraction based on a sliding window algorithm (Fig. 6a and Supplementary Fig. 2)

When the tiles are extracted from one WSI, the information about location and adjacent tiles is lost due to the limited fields-of-view. However, morphological information between adjacent areas is crucial for diagnostic decisions. Two typical tile extraction methods, the multiple ROI and sliding window methods, have been widely used to overcome this problem^9^. Although the multiple ROI method is faster than the sliding window because of its low redundancy, the sliding window method has the following advantages. First, the redundancy in the sliding window method assists data augmentation, an essential pre-processing step in a deep learning approach. Second, this method can overcome the limited field-of-view problem indirectly because the overlapping area depends on adjacent tiles. Finally, the overall accuracy can increase because the probability in the overlapping area is averaged during summation from the tile to the whole image. In this work, we choose the sliding window manner as the tile extraction method. Although the receptable maximum tile size is 512 × 512 pixels due to the limitation of our GPU memory size, we extracted a tile that is 1024 × 1024 pixels in size before the compression step. The stride is set to 256 pixels, horizontally and vertically.

### Z-axis compression based on principal component analysis (PCA)

**Figure 7:**
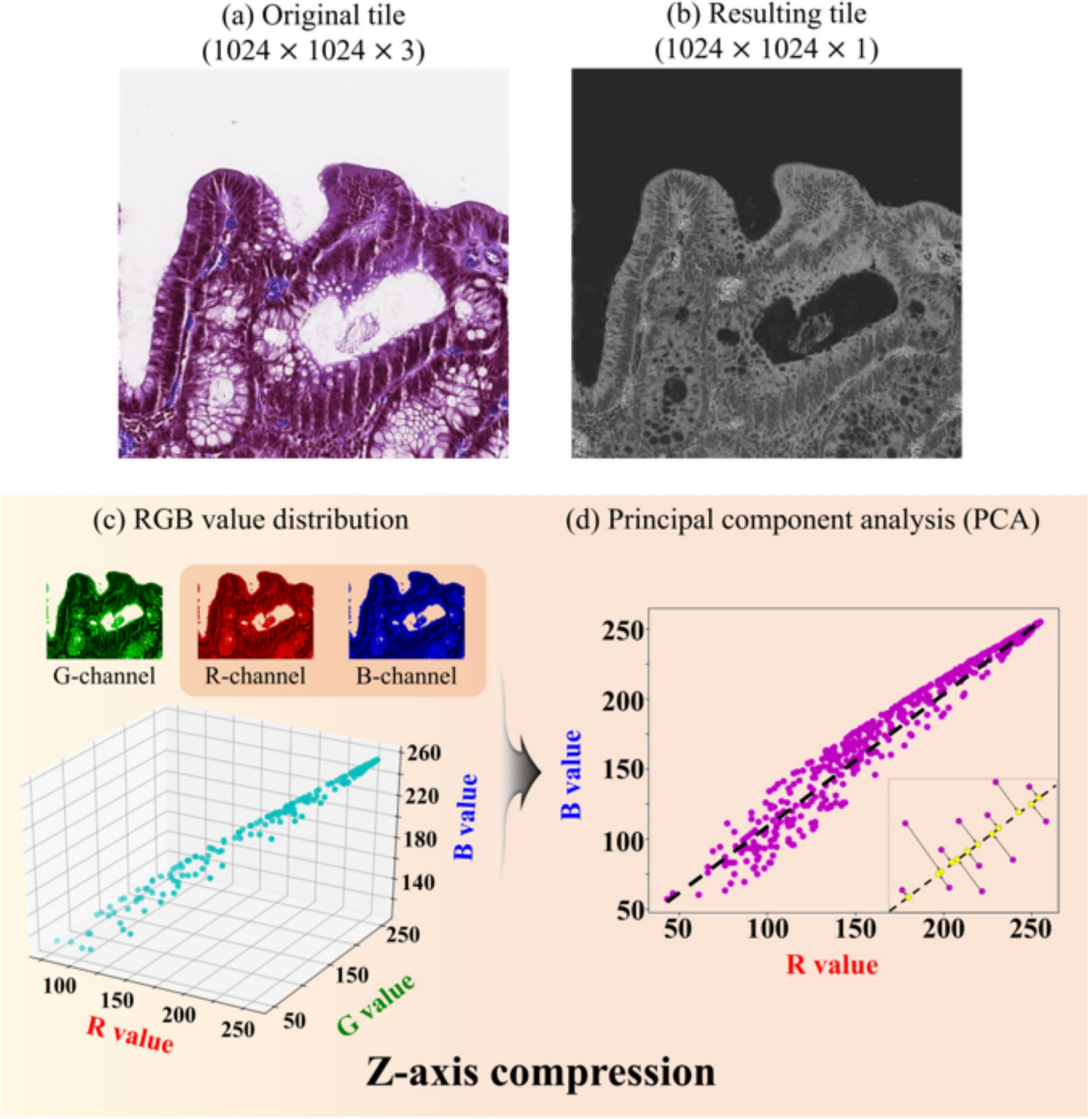
The principal component analysis (PCA) for z-axis compression. (**a**) The original tile composed of RGB-channels. (**b**) The resulting tile composed of single channel. (**c**) RGB value distribution in a tissue region. (**d**) The PCA for the R and B channels.

Pathologic images have three red (R), green (G), and blue (B) channels (Fig. 7a). The correlation is high among each color (Fig. 7c). Color variation in the pathologic image is given by H&E staining, which dyes the cell nuclei blue, and dyes the extracellular matrix and cytoplasm pink. Therefore, z-axis compression was applied only to the R and B channels in the tissue region. First, Otsu algorithm were applied to extract the RGB values at tissue region, and then the G values were removed^48^. PCA was applied to maximize the variation between the R and B values and to minimize the mean squared error (Fig. 7d)^28^. This process reduces the image dimensionality and results in background reduction, widely used in histopathology (Fig. 7b). The PCA algorithm is described in detail in Supplementary Table 3.

### Training neural networks in the compressed-domain (x- and y-axis compression)

After the image depth compression (z-axis), discrete wavelet transform (DWT) was performed on each tile to compress the information along the x- and y-axis^49^. Haar wavelet is usually used to extract texture feature^36,38,50^. So, we decided 2D DWT based on Haar wavelet and its sub-band was calculated using the following Eqs. (1)–(4):

1 $$ W_{\psi }^{A} (j,m,n) = \frac{1}{{\sqrt {MN} }}\sum\limits_{x = 0}^{M - 1} {\sum\limits_{y = 0}^{N - 1} {f(x,y)\psi_{j,m,n}^{A} } } , $$

2 $$ W_{\phi }^{V} (j,m,n) = \frac{1}{{\sqrt {MN} }}\sum\limits_{x = 0}^{M - 1} {\sum\limits_{y = 0}^{N - 1} {f(x,y)\phi_{j,m,n}^{V} } } , $$

3 $$ W_{\phi }^{H} (j,m,n) = \frac{1}{{\sqrt {MN} }}\sum\limits_{x = 0}^{M - 1} {\sum\limits_{y = 0}^{N - 1} {f(x,y)\phi_{j,m,n}^{H} } } , $$

4 $$ W_{\phi }^{D} (j,m,n) = \frac{1}{{\sqrt {MN} }}\sum\limits_{x = 0}^{M - 1} {\sum\limits_{y = 0}^{N - 1} {f(x,y)\phi_{j,m,n}^{D} } } , $$

where $\left(x, y\right)$ is the coordinate of the input tile, $\left(m, n\right)$ is the coordinate of the output sub-band, ${\psi }_{j,m,n}^{A}\left(x, y\right)$ and ${\phi }_{j,m,n}^{i}\left(x, y\right)$ represent the 2D wavelet basis function of level *j*, ${W}_{\psi }^{A}$ describes an approximation of the original image called the LL (low-low) sub-band, and ${W}_{\phi }^{V}$,${W}_{\phi }^{H}$, and ${W}_{\phi }^{D}$ are high-frequency components whose directions are vertical, horizontal, and diagonal. We call this transformed domain a compressed domain^26^. These components are called the LH (low–high) sub-band, HL (high-low) sub-band, and HH (high-high) sub-band, respectively. Our proposed method using these compressed domain analyses has the following benefits. First, the image size is reduced (e.g., from 1024 × 1024 pixels to 512 × 512 pixels), but all needed information is retained to perfectly reconstruct the original image. After reconstruction, the ROI can be increased without losing information, which is proportional to the generalization performance. Second, the method is useful for classifying texture because the result of the 2D grey-level co-occurrence matrix (GLCM) in the wavelet domain can capture texture information from the wavelet sub-band according to the cancer grading^36^. We input all four DWT sub-bands in parallel to each separate segmentation model, U-Net++^51^. We used the DiceCE loss function combined the Dice coefficients and the cross-entropies ^52^. Each sub-band model took two NVidia Titan X GPUs. The total batch size was six for each GPU.

### Prediction from tiles to whole images using wavelet weighted ensemble (WWE)

**Figure 8:**
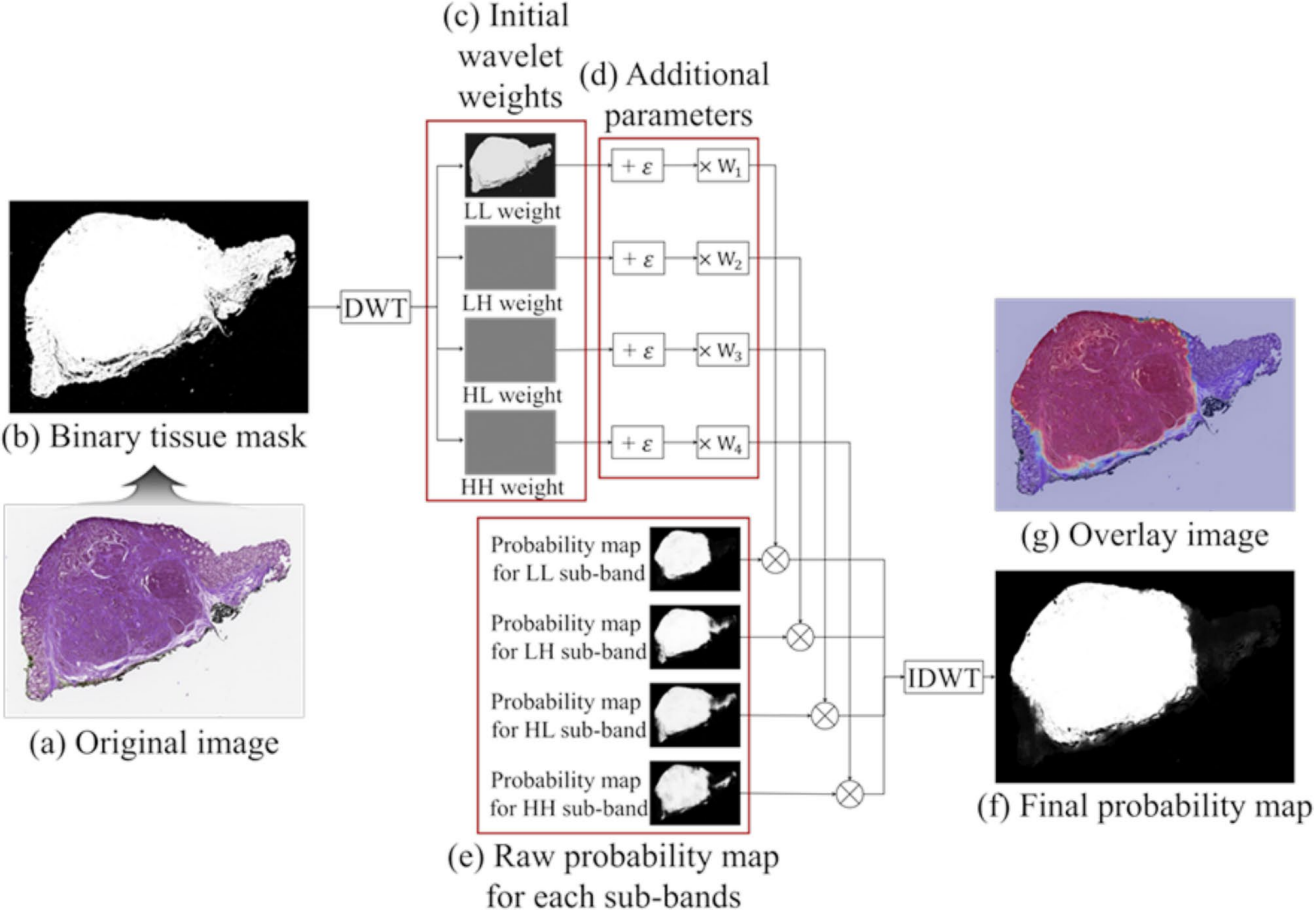
Schematic of wavelet weighted ensemble (WWE) for one low pass sub-band (LL sub-band) and three high pass sub-bands (LH, HL, and HH) based on discrete wavelet transform (DWT) and inverse discrete wavelet transform (IDWT). (**a**) Original image. (**b**) Binary tissue mask. (**c**) Initial wavelet weights. (**d**) Additional parameters. ε = 0.1, W_1_ = 2.1, W_2_ = 1.8, W_3_ = 1.8, and W_4_ = 3.0. (**e**) Raw probability map for each sub-band. (**f**) Final probability map. (**g**) Overlaid image.

The reconstruction process is described here. After producing a whole probability map for each sub-band, as shown in Fig. 6e, we applied ensemble learning based on wavelet weighted ensemble (WWE) to four trained neural networks for each sub-band (Fig. 8). Initially, a binary mask image (Fig. 8b) is obtained from the original image by using an Otsu algorithm (Fig. 8a)^48^. After a 2D wavelet transform based on the Haar wavelet, four wavelet sub-bands for the binary tissue mask were generated (Fig. 8c). We defined them as the wavelet weights, namely the LL weight, LH weight, HL weight, and HH weight. We added a small value, ε, to each wavelet weight, then multiplied it by their assigned weights (Fig. 8d). Lastly, we multiplied the weights by the corresponding probability map (Fig. 8e), and then applied an inverse discrete wavelet transform that also used the Haar wavelet to obtain a final probability map and overlay image (Fig. 8f,g). Parameters such as ${W}_{1}, {W}_{2}, {W}_{3},$ and ${W}_{4}$ were empirically determined. Ideally, if the same region of each sub-band has a probability of 1, the reconstruction probability of that region should also be 1 without those parameters. However, we gave the LL sub-band more weight (i.e., 1.8) because the LL sub-band has a basic characteristic of the original image. Then, ε was added to remove the zero terms. The ensemble method is expressed by the following Eq. (5):

5 $$\begin{aligned} {R}_{WWE} & =\frac{1}{\sqrt{MN}}\sum \limits_{x=0}^{M-1}\sum \limits_{y=0}^{N-1}{W}_{1}({Y}_{\psi }^{A}+\varepsilon ){R}_{A}(m,n){\psi }_{1,m,n}^{A} \\ & \quad +\frac{1}{\sqrt{MN}}\sum \limits_{i=H,V,D}\sum \limits_{m=0}^{M-1}\sum \limits_{n=0}^{N-1}{W}_{i}({Y}_{\phi }^{i}+\varepsilon ){R}_{i}(m,n){\phi }_{1,m,n}^{i},\end{aligned}$$

where ${\psi }_{1,m,n}^{A}\left(x, y\right)$ and ${\phi }_{1,m,n}^{i}\left(x, y\right)$ represent 2D wavelet basis functions of level 1, ${Y}_{\psi }^{A}$ describes an approximation of the binary tissue mask (LL sub-band weight), and ${Y}_{\phi }^{i}$ are high-frequency components (LH, HL, and HH sub-band weights) for the binary tissue mask whose directions are horizontal, vertical, and diagonal, respectively. ${R}_{A}$ and ${R}_{i}$ describe the probability map for each sub-band. ${R}_{WWE}$ is the final prediction result after wavelet weighted ensemble (WWE).

6 $$ \mathop {\arg \max }  \limits_{{W \in \Re^{4} }} \; f_{d} (R_{WWE}  \; (R_{A} ,R_{H} ,R_{V} ,R_{D} ;W)). $$

To optimize the weight parameters such as ${W}_{1}$, ${W}_{2}$, ${W}_{3}$, and ${W}_{4}$, we applied optimization that satisfied Eq. (6), where ***W*** = (${W}_{1}$, ${W}_{2}$, ${W}_{3}$, ${W}_{4}$) and ${f}_{d}(\mathrm{x})$ is the function that decides the average Dice score of x. The range of each parameter is from 0.3 to 3.0, with a step size of 0.3. For comparison, Supplementary Table 2 shows the average Dice scores for ${W}_{1}$, ${W}_{2}$, ${W}_{3}$, and ${W}_{4}$. We chose the parameter values as ${W}_{1}$ = 2.1, ${W}_{2}=1.8$, ${W}_{3}=1.8$, and ${W}_{4}=3.0$.

### Experimental setup

The qualities of the predictions were quantified by using the Dice score (Dice), pixel accuracy (Acc), and Jaccard score (Jac) as follows:

7 $$Dice=\frac{2\times {N}_{TP}}{{2\times N}_{TP}+{N}_{FP}+{N}_{FN}},$$

8 $$Acc=\frac{{N}_{TP}+{N}_{TN}}{{N}_{TP}+{N}_{TN}+{N}_{FP}+{N}_{FN}},$$

9 $$Jac=\frac{{N}_{TP}}{{N}_{TP}+{N}_{FP}+{N}_{FN}},$$

where ${N}_{TP},{N}_{TN}, { N}_{FP}, {\text{ and}} \, {N}_{FN}$ are the number of pixels for true-positive, false-positive, true-negative, and false-negative.

For the 39 WSIs test dataset, our proposed method was compared with the model in three ways: (1) Three condition of input image in spatial domain: standard (Tile size, 512 by 512 pixels; magnification, 20×), low resolution (Tile size, 512 by 512 pixels; magnification, 10×), and small ROI (Tile size, 256 by 256 pixels; magnification, 20×), (2) Four compressed data such as the LL, LH, HL, and HH sub-bands after PCA, and (3) using the weighted average ensemble (WAE) for each sub-band result after grayscale conversion and PCA and WWE for each sub-band result after grayscale conversion. The WAE is expressed as follows:

10 $$ R_{WAE} = \frac{{W_{1} R_{A} + W_{2} R_{H} + W_{3} R_{V} + W_{4} R_{D} }}{{W_{1} + W_{2} + W_{3} + W_{4} }}, $$

where ${R}_{A},$ ${R}_{H},$ ${R}_{V},$ and ${R}_{D}$ describe the probability maps for each sub-band, and ${R}_{WAE}$ is the final prediction result after the weighted average ensemble. ${R}_{A}$, ${R}_{H}$, ${R}_{V}$, and ${R}_{D}$ describe probability the maps for the LL, LH, HL, and HH sub-bands, respectively. We set the same weight values in WAE as those in WWE (${W}_{1}=2.1$, ${W}_{2}=1.8$, ${W}_{3}=1.8$, and ${W}_{4}=3.0$).

In order to verify the excellence of the proposed method, we progressed experiments after fivefold cross-validtaions as follows: (1) To compare average Dice, Jac and Acc according to each method, (2) To observe distribution of Dice, Jac and Acc according to all classes, (3) Check dice change of all classes according to low-frequency weight (${W}_{1}$) and high-frequency weight (${W}_{2}$, ${W}_{3}$, and ${W}_{4}$), (4) To compare sample images and its line profiles according to each method, (5) To compare with Dice of WWE, WAE, and the result in spatial domain according to threshold for tumor probability.

## Supplementary Information

Supplementary Information.

## References

[1] Yoshida H (2017). Automated histological classification of whole slide images of colorectal biopsy specimens. Oncotarget.

[2] Gertych A (2019). Convolutional neural networks can accurately distinguish four histologic growth patterns of lung adenocarcinoma in digital slides. Sci. Rep..

[3] Saha M, Chakraborty C, Racoceanu D (2018). Efficient deep learning model for mitosis detection using breast histopathology images. Comput. Med. Imaging Graph..

[4] Yoon H (2019). Tumor identification in colorectal histology images using a convolutional neural network. J. Digit. Imaging.

[5] Kainz P, Pfeiffer M, Urschler M (2017). Segmentation and classification of colon glands with deep convolutional neural networks and total variation regularization. PeerJ.

[6] Ho, D. J. *et al.* Deep multi-magnification networks for multi-class breast cancer image segmentation. *Comput. Med. Imaging. Graph.*. **88**, 101866 (2021). 10.1016/j.compmedimag.2021.101866 PMC7975990 33485058

[7] Komura D, Ishikawa S (2018). Machine learning methods for histopathological image analysis. Comput. Struct. Biotechnol. J..

[8] Tokunaga, H., Teramoto, Y., Yoshizawa, A. & Bise, R. Adaptive weighting multi-field-of-view CNN for semantic segmentation in pathology. In *Proceedings of the IEEE Computer Society Conference on Computer Vision and Pattern Recognition* 2019-June, 12589–12598 (2019).

[9] Chang HY (2019). Artificial intelligence in pathology. J. Pathol. Transl. Med..

[10] Thakur N, Yoon H, Chong Y (2020). Current trends of artificial intelligence for colorectal cancer pathology image analysis: A systematic review. Cancers.

[11] Bera K, Schalper KA, Rimm DL, Velcheti V, Madabhushi A (2019). Artificial intelligence in digital pathology—New tools for diagnosis and precision oncology. Nat. Rev. Clin. Oncol..

[12] Wang S, Yang DM, Rong R, Zhan X, Xiao G (2019). Pathology image analysis using segmentation deep learning algorithms. Am. J. Pathol..

[13] Nagtegaal ID (2019). The 2019 WHO classification of tumours of the digestive system. Histopathology.

[14] Bouteldja N (2021). Deep learning—Based segmentation and quantification in experimental kidney histopathology. J. Am. Soc. Nephrol..

[15] Kanava F, Toyokawa G, Momosaki S, Rambeau M, Kozuma Y (2020). Weakly-supervised learning for lung carcinoma classification using deep learning. Sci. Rep..

[16] Lu, M. Y. *et al.* Data-efficient and weakly supervised computational pathology on whole-slide images. *Nat. Biomed. Eng.* **5**, 555–570. (2021) 10.1038/s41551-020-00682-w PMC8711640 33649564

[17] Byun SS (2021). Deep learning based prediction of prognosis in nonmetastatic clear cell renal cell carcinoma. Sci. Rep..

[18] Laak J, Litjens G, Ciompi F (2021). Deep learning in histopathology: The path to the clinic. Nat. Med..

[19] Sirinukunwattana K (2020). Arti fi cial intelligence-based morphological fingerprinting of megakaryocytes: A new tool for assessing disease in MPN patients. Blood Adv..

[20] Kayid, A. M. *Performance of CPUs/GPUs for Deep Learning workloads* 25 (2018). 10.13140/RG.2.2.22603.54563.

[21] Crochiere RE, Rabiner LR (1981). Interpolation and decimation of digital signals—A tutorial review. Proc. IEEE.

[22] Franco M, Ariza-Araújo Y, Mejía-Mantilla JH (2015). Automatic image cropping: A computational complexity study Jiansheng. Imagen Diagnostica.

[23] Brunton, S. L. & Kutz, J. N. *Data Driven Science & Engineering—Machine Learning, Dynamical Systems, and Control*. 572 (2017).

[24] Carrillo-De-Gea, J. M., García-Mateos, G., Fernández-Alemán, J. L. & Hernández-Hernández, J. L. A computer-aided detection system for digital chest radiographs. *J. Healthc. Eng.* **2016**, (2016). 10.1155/2016/8208923 PMC5058572 27372536

[25] Liang, Y., Kong, J., Vo, H. & Wang, F. ISPEED: an efficient in-memory based spatial query system for large-scale 3D data with complex structures. In *GIS: Proceedings of the ACM International Symposium on Advances in Geographic Information Systems* 2017-Novem, (2017). 10.1145/3139958.3139961 PMC8110205 33977292

[26] Tang J, Deng C, Huang GB, Zhao B (2015). Compressed-domain ship detection on spaceborne optical image using deep neural network and extreme learning machine. IEEE Trans. Geosci. Remote Sens..

[27] Wang, J. Z., Nguyen, J., Lo, K. K., Law, C. & Regula, D. Multiresolution browsing of pathology images using wavelets. In *Proceedings/AMIA ... Annual Symposium. AMIA Symposium* 430–434 (1999). PMC2232834 10566395

[28] Zou H, Hastie T, Tibshirani R (2006). Sparse principal component analysis. J. Comput. Graph. Stat..

[29] Ma L (2019). Deep learning in remote sensing applications: A meta-analysis and review. ISPRS J. Photogramm. Remote. Sens..

[30] Falk T (2019). U-Net: Deep learning for cell counting, detection, and morphometry. Nat. Methods.

[31] Kim H, Baik JW, Jeon S, Kim JY, Kim C (2020). PAExM: Label-free hyper-resolution photoacoustic expansion microscopy. Opt. Lett..

[32] Baik JW (2021). Intraoperative label-free photoacoustic histopathology of clinical specimens. Laser Photonics Rev..

[33] Williams T, Li R (2018). An ensemble of convolutional neural networks using wavelets for image classification. J. Softw. Eng. Appl..

[34] Liu P, Zhang H, Lian W, Zuo W (2019). Multi-level wavelet convolutional neural networks. IEEE Access.

[35] Jafari-Khouzani K, Soltanian-Zadeh H (2003). Multiwavelet grading of pathological images of prostate. IEEE Trans. Biomed. Eng..

[36] Bhattacharjee S (2019). Multi-features classification of prostate carcinoma observed in histological sections: Analysis of wavelet-based texture and colour features. Cancers.

[37] Niwas, S. I., Palanisamy, P. & Sujathan, K. Wavelet based feature extraction method for Breast cancer cytology images. In *ISIEA 2010-2010 IEEE Symposium on Industrial Electronics and Applications* 686–690. 10.1109/ISIEA.2010.5679377 (2010).

[38] Shaukat A (2016). Automatic cancerous tissue classification using discrete wavelet transformation and support vector machine. J. Basic. Appl. Sci. Res..

[39] Cooley JW, Tukey JW (1965). An algorithm for the machine calculation of complex Fourier series. Math. Comput..

[40] Sorensen HV, Jones DL, Heideman MT, Burrus CS (1987). Real-valued fast Fourier transform algorithms. IEEE Trans. Acoust. Speech Signal Process..

[41] Bray F (2018). Global cancer statistics 2018: GLOBOCAN estimates of incidence and mortality worldwide for 36 cancers in 185 countries. CA Cancer J. Clin..

[42] Center MM, Jemal A, Ward E (2009). International trends in colorectal cancer incidence rates. Cancer Epidemiol. Biomark. Prev..

[43] Lambert R, Sauvaget C, Sankaranarayanan R (2009). Mass screening for colorectal cancer is not justified in most developing countries. Int. J. Cancer.

[44] Joseph DA (2016). Colorectal cancer screening: Estimated future colonoscopy need and current volume and capacity. Cancer.

[45] van den Bent MJ (2010). Interobserver variation of the histopathological diagnosis in clinical trials on glioma: A clinician’s perspective. Acta Neuropathol..

[46] Rubio CA (2006). The Vienna classification applied to colorectal adenomas. J. Gastroenterol. Hepatol..

[47] Japanese Society for Cancer of the Colon and Rectum (2019). Japanese classification of colorectal, appendiceal, and anal carcinoma: The 3d English edition [secondary publication]. J. Anus Rectum Colon.

[48] Otsu N (1979). A threshold selection method from gray-level histograms. IEEE Trans. Syst. Man Cybern.

[49] Rabbani, M. & Joshi, R. *An overview of the JPEG 2000 still image compression standard*. *Signal Processing: Image Communication* Vol. 17 (2002).

[50] Lee D, Choi S, Kim HJ (2019). High quality imaging from sparsely sampled computed tomography data with deep learning and wavelet transform in various domains. Med. Phys..

[51] Zhou, Z., Rahman Siddiquee, M. M., Tajbakhsh, N. & Liang, J. Unet++: A nested u-net architecture for medical image segmentation. *Lecture Notes in Computer Science (including subseries Lecture Notes in Artificial Intelligence and Lecture Notes in Bioinformatics)* 11045 LNCS, 3–11 (2018). 10.1007/978-3-030-00889-5_1 PMC7329239 32613207

[52] Isensee, F. *et al.* nnU-Net: Self-adapting framework for u-net-based medical image segmentation. *arXiv* (2018).

