# Supplementary material for: Deep learning-based histopathological segmentation for whole slide images of colorectal cancer in a compressed domain
Source: Sci Rep. 2021 Nov 18;11:22520. doi: 10.1038/s41598-021-01905-z (PMC8602325; doi:10.1038/s41598-021-01905-z)
Supplement: Supplementary file 1 — Supplementary Information. [file 41598_2021_1905_MOESM1_ESM.pdf]

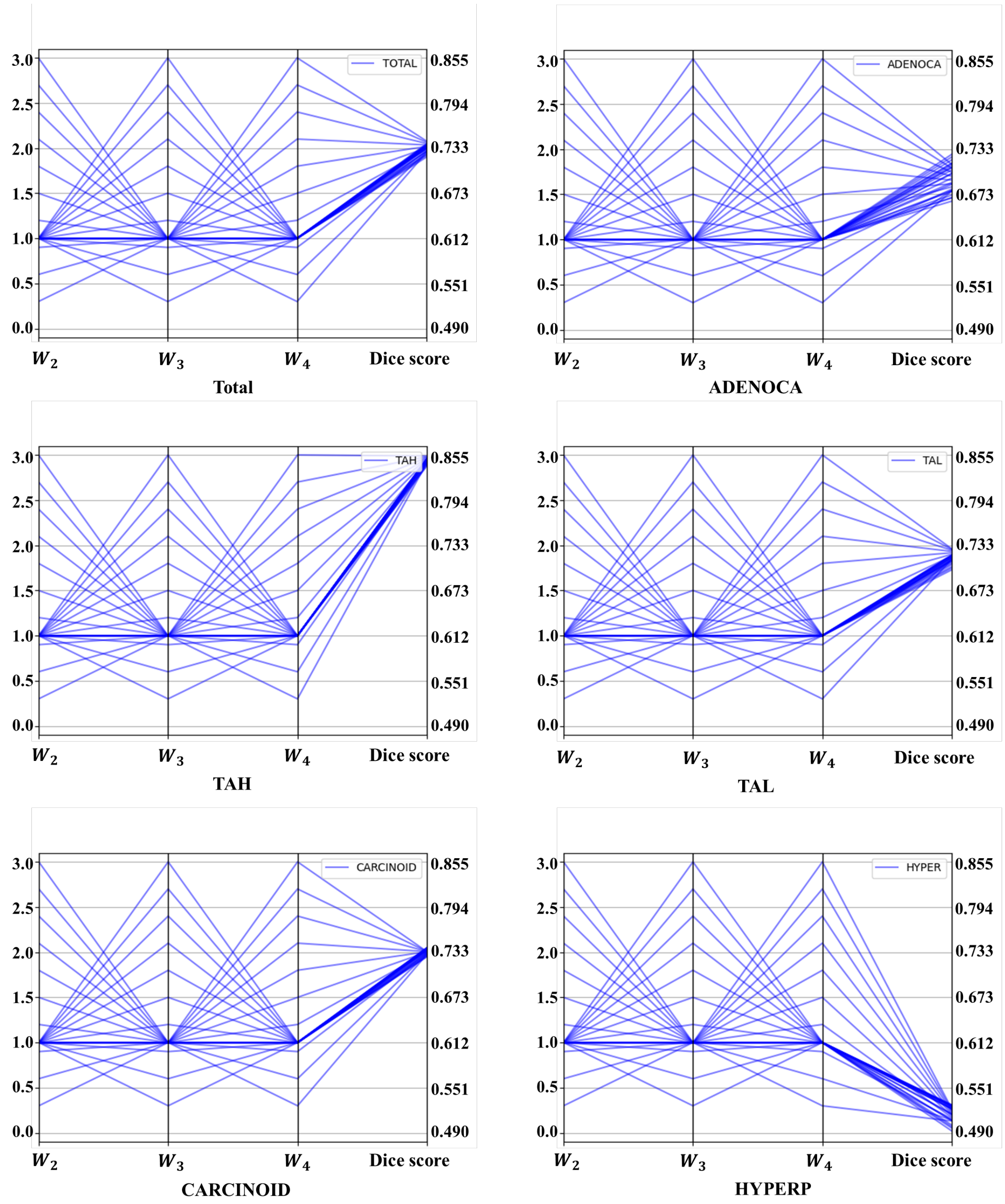

Supplementary Fig. 1. Parallel coordinates for high-frequency weights such as W_2, W_3, and W_4 according to the total class, ADENOCA, TAH, TAL, CARCINOID, and HYPERP.

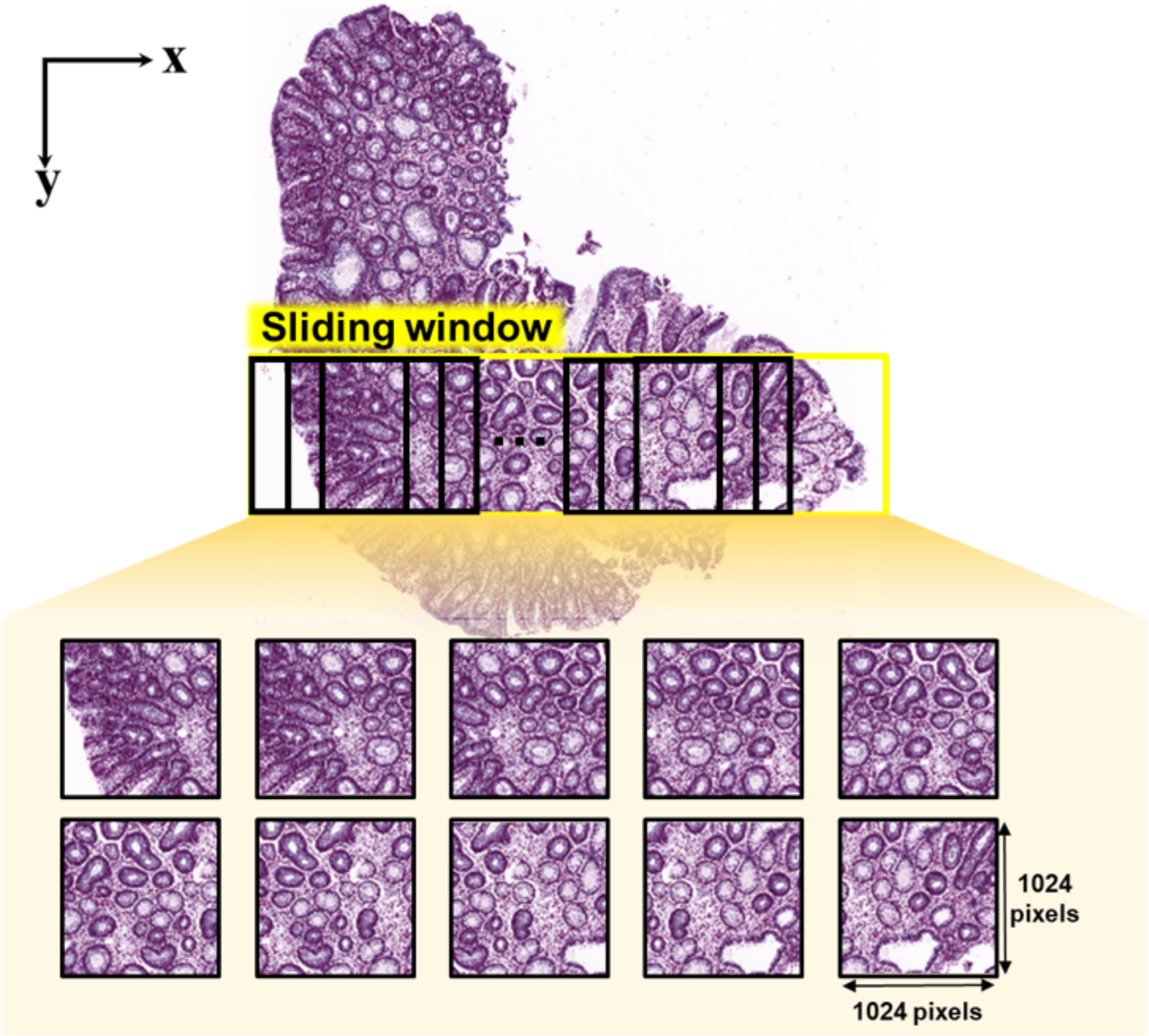

Supplementary Fig. 2. Tile extraction based on a sliding window (pixel size, 1,024 by 1,024 pixels; stride, 256 pixels).

Supplementary Table I. Class distribution for whole slide image (WSI). The tumor class is composed of adenocarcinoma (ADENOCA), high-grade adenoma with dysplasia (TAH), and low-grade adenoma with dysplasia (TAL), carcinoid (CARCINOID), hyperplastic polyp (HYPERP).

Tumor class	Train and validation	Test	Total
ADENOCA	44	5	49
TAH	96	11	107
TAL	122	14	136
CARCINOID	35	4	39
HYPERP	54	5	59
Total	351	39	390

Supplementary Table II. Comparison of average Dice scores for W_1, W_2, W_3, and W_4. The number in bold face type is relatively high value in the row.

	**0.3**	**0.6**	**0.9**	**1.2**	**1.5**	**1.8**	**2.1**	**2.4**	**2.7**	**3.0**
W_1	0.003 ± 0.004	0.300 ± 0.227	0.690 ± 0.172	0.774 ± 0.132	0.798 ± 0.123	0.805 ± 0.124	**0.806** ± **0.127**	0.805 ± 0.130	0.802 ± 0.134	0.800 ± 0.137
W_2	0.723 ± 0.156	0.726 ± 0.156	0.729 ± 0.154	0.733 ± 0.152	0.736 ± 0.151	**0.737** ± **0.149**	0.736 ± 0.149	0.735 ± 0.149	0.733 ± 0.150	0.732 ± 0.150
W_3	0.721 ± 0.157	0.724 ± 0.157	0.729 ± 0.155	0.733 ± 0.152	0.737 ± 0.149	**0.739** ± **0.147**	0.739 ± 0.146	0.738 ± 0.146	0.736 ± 0.147	0.735 ± 0.147
W_4	0.728 ± 0.155	0.729 ± 0.154	0.730 ± 0.154	0.731 ± 0.153	0.733 ± 0.153	0.736 ± 0.152	0.738 ± 0.151	0.740 ± 0.150	0.741 ± 0.149	**0.743** ± **0.149**

Supplementary Table III. The PCA algorithm for Z-axis compression

1: **Input**: 2-dimensional data set $X = \{\boldsymbol{x}_1, \boldsymbol{x}_2, \ldots, \boldsymbol{x}_N\}$ and the new dimensionality d = 1
2: Compute the mean $\overline{\boldsymbol{x}} = \frac{1}{N} \sum_{i=1}^{N} \boldsymbol{x}_i$
3: Compute the covariance matrix $\mathrm{Cov}(\boldsymbol{x}) = \frac{1}{N} \sum_{i=1}^{N} (\boldsymbol{x}_i - \overline{\boldsymbol{x}})(\boldsymbol{x}_i - \overline{\boldsymbol{x}})^T$
4: Find the spectral decomposition of Cov($\boldsymbol{x}$), obtaining the eigenvectors ξ_1, ξ_2 and their corresponding eigenvalues λ_1, λ_2. Note that the eigenvalues are sorted, such that $\lambda_1 \geq \lambda_2 \geq 0$
5: For any $\boldsymbol{x} \in \mathbb{R}^2$, its new lower dimensional representation is

$$\boldsymbol{y} = \left({\xi_1}^T (\boldsymbol{x} - \overline{\boldsymbol{x}}) \right) \xi_1 \in \mathbb{R}$$
